# Prioritization of healthcare systems during pandemics using Cronbach’s measure based fuzzy WASPAS approach

**DOI:** 10.1007/s10479-022-04714-3

**Published:** 2022-05-03

**Authors:** Muhammet Deveci, Raghunathan Krishankumar, Ilgin Gokasar, Rumeysa Tuna Deveci

**Affiliations:** 1 Department of Industrial Engineering, Turkish Naval Academy, National Defence University, 34940 Tuzla, Istanbul, Turkey; 2 Royal School of Mines, Imperial College London, London, SW7 2AZ UK; 3 Department of Computer Science and Engineeering, Amrita School of Engineering, Amrita Vishwa Vidyapeetham, Coimbatore, TN India; 4 Department of Civil Engineering, Bogazici University, 34342 Bebek, Istanbul, Turkey; 5 Department of Pediatric Hematology-Oncology, Faculty of Medicine, Istanbul University, 34093 Topkapı, Istanbul, Turkey

**Keywords:** Healthcare systems, Pandemics, Fuzzy sets, Multi-criteria decision making (MCDM), WASPAS, Cronbach’s measure

## Abstract

Pandemics are well-known as epidemics that spread globally and cause many illnesses and mortality. Because of globalization, the accelerated occurrence and circulation of new microbes, the infection has emerged and the incidence and movement of new microbes have sped up. Using technological devices to minimize the visit durations, specifying days for handling chronic diseases, subsidy for the staff are the alternatives that can help prevent healthcare systems from collapsing during pandemics. The study aims to define the efficient usage of optimization tools during pandemics to prevent healthcare systems from collapsing. In this study, a new integrated framework with fuzzy information is developed, which attempts to prioritize these alternatives for policymakers. First, rating data are assigned respective fuzzy values using the standard singleton grades. Later, criteria weights are determined by extending Cronbach´s measure to fuzzy context. The measure not only understands data consistency comprehensively, but also takes into consideration the attitudinal characteristics of experts. By this approach, a rational weight vector is obtained for decision-making. Further, an improved Weighted Aggregated Sum Product Assessment (WASPAS) algorithm is put forward for ranking alternatives, which is flexibly considering criteria along with personalized ordering and holistic ordering alternatives. The usefulness of the developed framework is tested with the help of a real case study. Rank values of alternatives when unbiased weights are used is given by 0.741, 0.582, 0.640 with ordering as $R_{1} \succ R_{3} \succ R_{2}$. The sensitivity/comparative analysis reveals the impact of the proposed model as useful in selecting the best alternative for the healthcare systems during pandemics.

## Introduction

Several outbreaks have happened throughout human history. The longest-lasting, most-repeated, and most-deadly diseases are the plague, smallpox, cholera, and Spanish flu (Akin and Gozel, 2020). Few phenomena have affected our communities and cultures as much as outbreaks of infectious diseases (Huremović, 2019). Globalization has also facilitated the exchange of information and experiences in those conditions (Akin and Gozel, 2020).

The recent outbreak of the coronavirus, which was announced first in the People’s Republic of China’s Hubei, has been seen in a lot of different countries (Velavan & Meyer, 2020). The WHO Emergency Committee declared a global health emergency on January 30, 2020 (Jee, 2020). Pneumonia incidents that were related to the Seafood Wholesale are analyzed through epidemiological studies. Respiratory samples into human epithelial, Vero E6, and Huh7 cell lines resulted in the isolation of a novel respiratory virus, identified as a novel coronavirus related to SARS-CoV based on genome analysis (Ciotti et al., 2020). The case execution rate changes regularly and can be recorded in real-time on the website provided by Johns Hopkins University and other forums. According to the COVID-19 report of Johns Hopkins University on 10^th^ December 2021, the number of confirmed cases worldwide has reached 268,682,986 while the total number of death is over 5,290,949 and more than 242,027,157 have recovered (https://coronavirus.jhu.edu/).

Massive demand for addressing the COVID-19 epidemic overwhelmed all healthcare workers and the hospital supply system. The provision of certified protective gear to hospital workers is critical in preventing workers' exposure and infection. Protective gear is rarely used in clinical settings. So, it is not in bulk supply (Cao et al., 2020). Every minute a doctor, nurse, or other staff member serves a COVID-19 patient and they endanger their health. Insufficient social distance is one of the adjustable risks if handled by the deployment of regulated physical separation, increased availability of protective gear, and suitable recommendations, would drastically lower infection rates and prevent fatalities (Ehrlich et al., 2020). To promote security, management techniques should be implemented as soon as possible (Felice et al., 2020). It is critical to use effective techniques to manage the wellness of health care workers and patients (Ehrlich et al., 2020).

Epidemics present many obstacles in the delivery of healthcare (Chauhan et al., 2020). New and engaging systems are necessary to serve the crucial requirements of COVID-19 patients and other individuals who need healthcare services. New technologies give additional choices in this regard (Carrabba et al., 2020). Even though the eventual COVID-19 approach will be multidimensional, it is one of the successful methods to employ existing technology to support efficient service delivery by decreasing the risk of direct person-to-person spreading (Smith et al., 2020; Zhou et al., 2020). The AAP (2020) initiated a COVID-19 crisis preparation and action telementoring service. The COVID-19 Project Extension for Community Health Care Outcomes approach is a telementoring system that links a diverse team of experts with primary care physicians in local areas.

Optimization strategies can find the best potential solution to a defined problem. Optimization strategies are used in various fields of study to find solutions that maximize or minimize particular study characteristics. However, its scope is broad, as are the approaches used to tackle the healthcare systems during pandemics (Alonso et al., 2020). Contact tracing is one of the most COVID-19 control strategies. Isolation of sick people, contact tracing, and isolating all their contacts will be required to reduce community spread. The outbreak’s growth rate can be slowed by lowering contact rates. Controlling contact rates is critical to outbreak management (Rocklöv & Sjödin, 2020). Avoiding enormous groups and busy public locations, as well as keeping at least 6 feet of space between oneself and others, will keep one from getting COVID-19, which is known as social distancing (Desai & Patel, 2020). Using optimization tools will probably prevent increasing the number of patients. Science provides the key to improving the quality of life for everyone, from infants to older people. Technological improvements are a catalyst that can profoundly revolutionize healthcare and the practice of medicine. Therefore, any technology that reduces the loss of human life and improves life quality has a value worth. Furthermore, the healthcare industry must face the problem of balancing cost conservation with the maintenance of desired patient outcomes (Park & Jayaraman, 2003). To cope with the collapses during pandemics, decision-makers should consider all optimization alternatives.

### The motivation of the proposed model

Based on the review works from Zavadskas et al. (2014), Simic et al. (2017) and Mardani et al., (2017), it can be inferred that (i) fuzzy set is a simple, elegant, and popular preference style used by researchers for decision-making and (ii) WASPAS is also a simple and powerful ranking method used by many researchers in solving practical decision problems. Further, these reviews show that (i) WASPAS could not capture the nature of criteria during ranking, and (ii) personalized ranking based on the data from a specific expert is lacking in the existing models.

These inferences and challenges motivated authors to put forward an integrated framework with fuzzy data. Besides the challenges identified in the existing ranking method (from Mardani et al., 2017), authors could also infer that weights of experts must be methodically determined, which mitigates biases and inaccuracies (Kao, 2010). Motivated by the claim from (Kao, 2010), the authors put forward Cronbach’s measure for weight calculation. Contributions of the work can be summarized as follows:

- Simple yet elegant preference style is considered in this study. Fuzzy numbers are adopted for rating alternatives based on competing criteria. Likert scale ratings are transformed to fuzzy values to avoid subjective randomness in the decision process,
- Cronbach’s measure is put forward for determining the importance (weights) of each competing criterion methodically. The measure has the ability to determine the consistency of experts in rating criteria by adopting comprehensive similarity estimates in the formulation. Furthermore, the proposed measure can effectively capture the hesitation of experts during the rating elicitation process,
- The classical WASPAS (Zavadskas et al., 2012) approach is improved in two folds, namely *(i)* the method can flexibly take into consideration the nature of criteria and *(ii)* perform personalized ordering of alternatives based on individual´s data, along with holistic ranking order.
- Finally, the developed model is tested using a real case study for healthcare system evaluation and sensitivity analysis, followed by a comparative study from the application and method perspectives are performed that reveals the merits and shortcomings of the framework.

The objective of this study is to prioritize healthcare system alternatives during pandemics using the proposed multi-criteria decision making (MCDM) methodology. These alternatives are evaluated by the experts under 4 main criteria aspects, which contain 12 sub-criteria.

The rest of this study is organized as follows. Section 2 reviews the existing studies related to healthcare system studies during pandemics. The case study, criteria, and alternatives are defined in Sect. 3. Section 4 presents a novel MCDM methodology. The experimental results are presented in Sect. 5. Section 6 provides the results and discussion of the ranking of alternatives. The managerial policy implications and limitations and conclusions are given in Sects. 7 and 8.

## Research background

The World Health Organization declared COVID-19 as a pandemic in March (Barranco & Ventura, 2020). The pandemic has affected huge numbers of people, who are either ill or dying because of the disease’s spread. The most common symptoms of the virus are fever, cold, bone pain, and breathing difficulties, which can develop into pneumonia (Haleem et al., 2020). Hospitals in many countries got overcrowded, and COVID-19 led to overflowing Intensive Care Units. Although most people stayed at home, healthcare workers worked during the lockdown. The time between the onset of illness and death ranges from 6 to 41 days, with a 14-day average (Barranco & Ventura, 2020). Pandemics have a wide range of effects on our lives. One of the most important aspects is healthcare. While patients with other diseases were being neglected, doctors and other staff were at risk. As a result, healthcare workers and society faced many challenges during pandemics, both in terms of health and economics (Haleem et al., 2020).

The handling of COVID-19 patients who require immediate care is one of the most crucial aspects of this problem (Rahmatizadeh et al., 2020). Using technological (such as wearable) equipment for monitoring patients and diagnostics to reduce visit durations can prevent collapsing during pandemics. Wearable Health Devices (WHDs) are technological tools that allow for constant mobile monitoring of human signs in daily life or a medical setting. WHDs provide those with the benefit of minimal discomfort and interruption by general human actions (Dias et al., 2018). Equipped with the information, the doctor may provide prompt and safe treatment based on solid decision-making, ensuring the patient’s survival and establishing an effective healthcare delivery system. Mobile information infrastructure or monitoring systems should adapt to the individual’s requirements to take advantage of advances in telemedicine (Park & Jayaraman, 2003). The body has a variety of physiological indications that may be evaluated, ranging from electromagnetic to biochemical. Human bio-signals can be collected and then used to better understand the body’s health condition and responses to environmental factors. It is essential to grasp the primary bio-signals that contribute to a better human body in health diagnosis before comprehending how the signs are formed and how they can be obtained using wearable sensors and devices (Dias et al., 2018). The goal was to pique people’s interest in their health state while also increasing care quality and using new technology capabilities. These gadgets promote collaboration among various scientific areas, including biomedical technologies, micro and nanotechnologies, and information and communication technologies (Dias et al., 2018). Since current virus testing and vaccines take time to become ready, there is a demand for more effective disease diagnosis and monitoring of individual and population health, with which wearable sensors could help. While the utility of this technology has been used to connect physiological parameters to daily activities and human function, its application to predicting the occurrence of COVID-19 remains a necessity. Wearable device users may be alerted when changes in their measurements match connected with COVID-19 when used with predicting platforms (Seshadri et al., 2020). The advancement of integrated sensor technology has enabled precise remote measurement of several physiologic indicators, many of which are medically valuable in monitoring the progression of the disease as a viral illness (Seshadri et al., 2020). During pandemics, wearing garments can lower the number of patients and assure social distancing.

It is conceivable to create an Intelligence framework for predicting if a person is at increased risk of getting a health emergency or not depending on particular data from the person (Rahmatizadeh et al., 2020). Supply Chain Network Design (SCND) is a strategic move that is critical to a supply chain's success. SCND involves the facility's locations, quantities, and limitations (Devika et al., 2014). A study (Beheshtifar & Alimoahmmadi, 2015) uses a multiobjective nonlinear model to establish a sustainable supply chain system in the health industry. The purpose is to reduce inequities in access to health care services by using an algorithm. Developing software products that can automatically classify the danger of a disaster in various individuals is viewed as a method for having individualized therapy (Badnjevic et al., 2018). In addition, several monitoring methods have tracked the onset of respiratory infections (Ghassemi et al., 2015). In a changing situation, the function of artificial intelligence must be novel. Otherwise, the patient-care or medical judgment processes will be disrupted (Ruiz et al., 2019).

Specifying days for handling chronic diseases also can avert spreading viruses. Pandemics are complicated incidents. Because of the risk of infection during the COVID-19, patients canceled or delayed many visits (Wright et al., 2020). Facing equity problems is possible during pandemics (World Health Organization) but in today’s world, we can do a lot of things to create a fair environment. The COVID-19 epidemic is hastening the shift away from in-person outpatient services. Online and asynchronous healthcare systems by outpatient providers became the new standard almost instantly. Health systems are reinventing how they can effectively manage chronic diseases in a COVID-19 period. Health-care systems will harness the power of virtual communities to encourage healthy habit change (Mirsky & Horn, 2020). The first process in combating chronic disease in the COVID-19 period is the creation of an actual clinical registration system to identify and supervise high-risk and rising-risk patients. A clinical hypertension registry, which continuously monitors blood pressure control, tracks medication management prescriptions and multimorbidity, and analyzes appointment data across a whole patient population, is easily built or purchased by health systems (Mirsky & Horn, 2020). Regulation in date and online healthcare systems can stop the chaos.

Subsidy for the staff for shift days based on the number of incidents can optimize the situation during pandemics. A simplified example of the importance of economic freedom is resilience in pandemics. A pandemic changes the demand for certain goods and services, such as personal protection equipment, vaccine investigations, hospital ventilation systems, food delivery, and so on. As a result, consumers’ optimal consumption bundles change, requiring the quantity supplied of such goods to adjust (Candela & Geloso, 2021). Although decreasing the cost was the primary goal of distribution networks in general, organizations are accountable for the wellness and safety of all workers and the general public (Devika et al., 2014). Wage subsidy strategies that are well-designed can help to mitigate the economic effects of the pandemics. The primary policy goal of wage subsidy programs is to encourage the staff. Subsidies in pandemics are crucial to keeping employees in operation. Even lower subsidies can be beneficial during pandemics. Policymakers should understand the severity of pandemics (Law, 2020).

The following studies may also be useful for supply chain management and solving other problems in the healthcare industry: sustainable closed-loop supply chain network of face masks during the COVID-19 pandemic (Tirkolaee et al., 2022), sustainable operational management (Goli et al., 2021), allocation and routing model for relief vehicles (Goli et al., 2020), sustainable supply chain network (Pahlevan et al., 2021), and robust optimization for a multi-objective product portfolio problem (Goli et al., 2019).

## Problem definition

COVID-19 has presented a challenge to healthcare systems worldwide. Lack of transmission certainty, limitations in physical healthcare system facilities and equipment, and workforce shortages cause dynamic resource deployment to meet rapidly changing care needs. Healthcare systems need to invest in hardware, training, and provider infrastructure before the pandemic (Baumgart, 2020). While everyone faces these challenges, policymakers should evaluate all alternatives to prevent collapses during pandemics. In this study, decision-makers are supposed to consider taking actions to prevent collapses during pandemics. There are three distinct policies to prevent collapses.

### Definition of alternatives

There are some alternatives for decision-makers to prioritize to use optimization techniques effectively during pandemics. Three distinct policies are selected in this study.

*A*_*1*_*: Using technological (such as wearable) devices for monitoring and diagnosis of patients to minimize the visit durations:* The latest quandary confronting healthcare systems around the world is how to maintain the capability to fulfill not only those infected with COVID-19 but also trauma patients and those suffering from other acute and chronic diseases while protecting physicians, nurses, and other aligned health personnel. Healthcare systems around the world are turning to telemedicine. The widespread adoption of telemedicine shows its effectiveness as a tool for so-called social distancing in medical or other settings (Bashshur et al., 2020). There are various types of WHDs available, including smart bracelets that help track patient vital signs. Medical deterioration is effectively addressed by tracking vital signs. The requirements of the wearable system should be analyzed before designing. A smart bracelet can provide a patient’s vital signs. This can be accomplished by administering the survey designed to collect data from health experts (Naji et al., 2021).

*A*_*2*_*: Specifying days for handling chronic diseases:* To create a safe environment for patients who have chronic diseases, it is essential to specify days and provide online healthcare systems. Ambulatory care providers’ use of online and asynchronous healthcare systems became the new norm during pandemics (Mirsky & Horn, 2020). The COVID-19 has raised awareness of the critical necessity to prevent the spread of infectious and chronic diseases. To make chronic disease prevention at the highest for all individuals, the healthcare community needs to adopt a new attitude (Kmetik et al., 2021).

*A*_*3*_*: Subsidy for the staff for shift days based on the number of incidents:* Policymakers should understand the needs of pandemics and motivate the staff with subsidies. COVID-19 has caused lockdowns and other worker-impacting measures, resulting in a massive productivity shock. Some countries have implemented policies to avoid job losses to mitigate the impact of that shock, also subsidizing payrolls and providing financial support to companies that dedicate to ensuring the employees (Céspedes et al., 2020).

### Definition of aspects and criteria

#### Cost aspect

*C*_*1*_*: Necessary arrangements in the facilities:* Increased urgent needs in facilities have been seen during pandemics. With intense contact tracing, the people could resume normal life almost immediately. The virus could only persist in untested individuals, and contact tracing would frequently lead to them (Peto, 2020). Not each facility has such an opportunity. Government should contribute to those facilities.

*C*_*2*_*: Personnel cost:* The financial effects of outbreaks are significant (Rasul, 2020). Even when the number of pertussis cases is low, bacteremia immunization outbreaks incur significant costs for health facilities. The cost-effectiveness of prevention strategies for bacteremia immunization outbreaks, such as vaccination of healthcare workers, should be assessed (Baggett et al., 2007).

*C*_*3*_: *Cost of the resources used in a pandemic:* The development of efficient early detection methods is vital for future planning, and it might be expensive when cost overtime is considered (Morganstein et al., 2017). Social distancing and quarantine procedures have been implemented to slow the spread of COVID-19. The recovery field should be proactive instead of reactive to provide services that mitigate the effects of social distancing. When developing new software or shifting an existing program to a new delivery feature, such as telehealth, the cost is an important concern. The permission for the telehealth platform was the most expensive part of the development phase. When developing new programs, selecting a telehealth platform may be an easy target for cost reduction. There are hardly any expensive options available; however, features must be considered (Middleton et al., 2020).

#### Health aspect

*C*_*4*_*: Spread of the pandemic in the hospital:* Cross-contamination between patients is a risk of the pandemic in the medical environment (Ip et al., 2020). With the effective use of optimization tools, such a situation cannot be observed. Because of a lack of PPE, many infrastructural suggestions include no or reduced PPE for the care of the asymptomatic patient with no recognizable risk factors (Ip et al., 2020).

*C*_*5:*_* Delayed scheduled healthcare:* Early diagnosis is essential for COVID-19 prevention and control (Wu et al., 2020). Implementing efficient early detection systems is critical for long-term preparation. Resilience to committing public funds needed to ensure early detection is common, as one common behavioral response to pandemics is to believe that delayed awareness is sufficient (Morganstein et al., 2017).

*C*_*6*_*. The health condition of the personnel:* COVID-19 puts additional strain on doctors and the healthcare system as a whole (Galbraith et al., 2021). Researches show that such strain raises the risk of mental distress for doctors. During such a dangerous public health event, a 5G network is worth considering as a viable strategy (Wu et al., 2020).

#### Efficiency and capacity aspect

*C*_*7*_*. The capacity of the personnel:* Inadequate worker capacity caused significant concern in the nursing community and led to health care personnel citing a right to refuse to work (Tzeng, 2004). Anxiety among health care workers can be reduced by ensuring proper healthcare worker capacity during emergencies such as epidemics (Ruderman et al., 2006).

*C*_*8*_*: The efficiency of the personnel:* Guaranteeing that health care personnel are educated about infectious diseases, that they are provided with and educated to use personal protective equipment, and that they have access to medical and psychiatric resources can help reduce the negative influences on this community (Morganstein et al., 2017).

*C*_*9*_*: The capacity of the resources:* The supply for treatment can rapidly outstrip a community’s medical response capacity. Planning for the mental and physiological responses that accompany the surge in health demand, community responses to scarcities, and early treatment approaches following pandemic identification is a critical component of public health preparation (Morganstein et al., 2017). Many doctors, in particular, were unfamiliar with lung ultrasound. Personal protective equipment and ultrasound devices machinery were also inadequate (Wu et al., 2020). With the help of technological devices, resources will be adequate for everyone else.

*C*_*10*_*: Technology and innovation use:* Although global threats persist, advances in technology and health care have enabled more early recognition, evaluation, and treatment (Morganstein et al., 2017). Effective artificial intelligence and telehealth systems have emerged, which could play a significant role in the current outbreak. Tele Ultrasound, including both synchronous and asynchronous modes, can be used over long distances. Under a 5G network, machine tele ultrasound, which is controlled by a skilled sonographer in the remote area, can operate in continuous mode (Wu et al., 2020).

*C*_*11*_*: Physical capacity:* During pandemics, the physical capacity of hospitals and also intense care units in hospitals are not enough. Technological improvements help reduce the number of patients in hospitals. The public health care system should be ready for surges in healthcare demand, particularly in the early stages of a pandemic. People and their families should be guided to improve their psychological health. The demand for care can quickly outstrip a community’s healthcare response capacity (Morganstein et al., 2017).

#### Social aspect

*C*_*12*_*: Equity among the patients:* The number of patients during the pandemic increased dramatically, causing problems with a lack of medical resources and ultrasound specialists (Wu et al., 2020). Not only for COVID-19–related care but also for patients with chronic diseases, health systems have embraced telemedicine at considerable speed (Nouri et al., 2020). Without assertive initiatives to maintain equity, the existing widespread adoption of telehealth may exacerbate differences in health care access for vulnerable communities with low digital skills or access (Nouri et al., 2020). Government should provide a gained education and training for those.

*C*_*13*_*: Demographics´ access to uninterrupted healthcare:* Everyone has equal access to the healthcare system. During pandemics, patients’ inequity can be seen. It is possible to reduce it with technological advancements. Availability of medicine is an important element of healthcare systems. Undisrupted access to medicine is much needed and crucial for the population's health (Akande-Sholabi & Adebisi, 2020).

*C*_*14*_*: Inconvenience of the patients while getting a health service:* The COVID-19’s serious interruptions have compelled a redistribution of resources to meet hospital systems' existing service needs during the pandemic (Nuñez et al., 2020). COVID-19 has sparked widespread concern in the global public health community. Significant decreases in emergency trauma service visits, workplace injuries, car crashes, and hospital admissions were discovered (Nuñez et al., 2020).

## Methodology

**Fig. 1:**
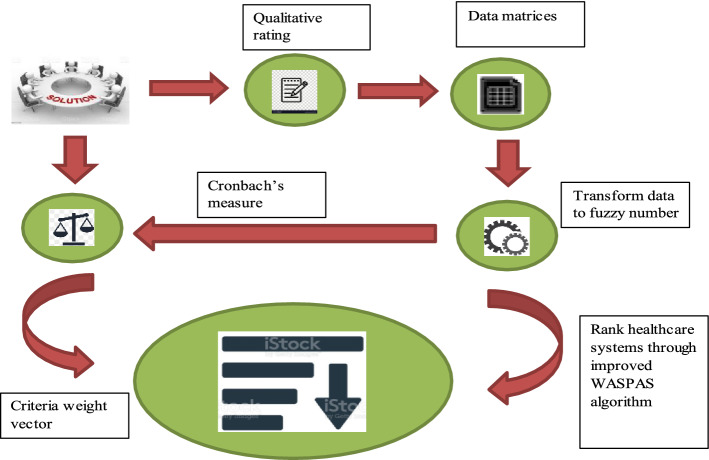
Proposed model for prioritization of healthcare systems with fuzzy information

In this section, the steps of the proposed model are presented. The diagram depicted in Fig. 1 is the proposed decision framework that intends to prioritize healthcare systems by using fuzzy data. $t$ matrices of order $r \times q$ are obtained with a linguistic rating that is converted to its respective fuzzy values for the decision process. Also, $t$ vectors of $1 \times q$ order are obtained for criteria weight calculation. Cronbach’s measure is extended to fuzzy context by incorporating attitudinal characteristics of experts in its formulation, which supports the rational estimation of weights and effective capturing of the consistency of the data. A weight vector of $1 \times q$ is obtained, which is further fed as input for the ranking algorithm along with the decision matrices from experts. By using the novel algorithm, personalized ranking order is obtained along with a holistic ordering of alternatives that formulates to $t$ personalized $1 \times r$ rank vectors and a fused rank vector of $1 \times r$ that is used for ordering the alternatives.

### Criteria importance through Cronbach’s measure

Determination of the relative importance of criteria is an important phase in decision-making. Practically, multiple criteria have unbiased weight values associated with them and direct weight assignment is subjected to biases and inaccuracies (Kao, 2010). To mitigate the problem, researchers have methodically determined weights, which can be broadly classified as weight determination with partial information and unknown information.

Optimization models (Krishankumar et al., 2021; Sivagami et al., 2021) are put forward in the former category and the system incurs overhead for obtaining partial information on each criterion. When experts are unable/unwilling to express their views on the importance of criterion, the methods from the latter category are put forward, such as entropy (Zheng et al., 2020; Mahmood and Ali, 2020), analytical hierarchy process (Anbuudayasankar et al., 2020), best–worst approach (Rezai, 2015; Pamucar et al., 2020), and so on. These methods determine weights methodically, but are complex and are unable to capture the hesitation of experts during choice sharing. Moreover, these methods cannot capture interactions among criteria.

Motivated by these challenges, authors present a novel approach that takes into consideration criteria interactions and hesitation of experts comprehensively by extending Cronbach’s measure to fuzzy context. The approach intends to alleviate the issues mentioned above and the stepwise procedure is given below:

*Step 1:* Form an opinion matrix with order $t \times q$ by considering linguistic scales for rating. $t$ experts share her/his preferences on $q$ criteria.

*Step 2:* Assign fuzzy numbers to each term and form a distance vector using the city block distance measure criteria-wise. This attempts to capture the variability in the rating given by experts. Equations (1)- (2) apply to capture the relationship between preferences, which in turn captures the homogeneity in the experts’ characteristics.

1 $$ d_{j} \left( {a,b} \right) = \mathop \sum \limits_{{a,b \in \frac{{t\left( {t - 1} \right)}}{2}}} \left| {\mu_{a} - \mu_{b} } \right| $$

2 $$ \rho_{j} \left( {a,b} \right) = 1 - d_{j} \left( {a,b} \right), $$

where $a$ and $b$ are any two experts, $d_{j} \left( . \right)$ is the distance measure pertaining to the criterion $j$, and $\rho_{j} \left( . \right)$ is the similarity measure associated with criterion $j$.

*Step 3:* Apply Eq. (3) to determine the Cronbach’s coefficient for each criterion that yields a vector of $1 \times q$ order.

3 $$ \beta_{j} = \frac{{t.\overline{{\rho_{j} \left( {a,b} \right)}} }}{{1 + \left( {t - 1} \right).\overline{{\rho_{j} \left( {a,b} \right)}} }}, $$

where $\overline{{\rho_{j} \left( {a,b} \right)}} \overline{{\rho_{j} \left( {a,b} \right)}}$ is the mean of similarity values calculated for criterion *j*.

*Step 4:* Normalize the coefficient values to obtain weight vectors of $1 \times q$ order that is of unit interval range and sum yielding unity.

4 $$ w_{j} = \frac{{\beta_{j} }}{{\mathop \sum \nolimits_{j} \beta_{j} }}, $$

where $w_{j}$ is the weight of criterion $j$.

### Alternatives ranking through improved WASPAS

The ranking is another key aspect of decision-making. In this phase, alternatives are ordered that enable experts to decide on the suitability of the options for the process under consideration. WASPAS (Zavadskas et al., 2012) is a ranking approach that considers weighted sum and weighted product formulation in a linear combination fashion for determining the order the alternatives. Evidently, the method gained popularity in the decision-making models owing to its simplicity and elegance in understanding the ranking process. Chakraborty et al. (2015) presented the implementation of WASPAS as a decision tool by provide the stepwise calculation of parameters. Karabasevic et al. (2016) put forward an integrated framework under uncertainty context with SWARA for weight assessment and WASPAS for ranking personnel in a firm. Mardani et al. (2017) prepared a meta-analysis of WASPAS and showed its usefulness in diverse practical applications of decision-making such as medical, supply chains, environment, and so on. Tuş and Aytaç Adalı (2019) gave a hybrid model with CRITIC for weight calculation and WASPAS for assessing software related to attendance of time in a firm. Turskis et al. (2019) extended WASPAS to fuzzy context for assessing critical information infrastructures towards EU sustainable development goals. Mishra et al. (2019) extended WASAPS to hesitant fuzzy context for green supplier evaluation with unknown weight information. Krishankumar et al. (2019) put forward a new framework with probabilistic linguistic data for green supplier evaluation by using variance method and WASPAS method for weight calculation and ranking process. Pamucar et al. (2020) proposed a fuzzy based framework with EBWA and WASPAS for weight determination and ranking of access modes in airport. Yücenur and Ipekçi (2021) came up with a framework for marine current plant location under the uncertainty context by combining SWARA and WASPAS for weight calculation and ranking process. Bouchraki et al. (2021) put forward a new model with fuzzy AHP and fuzzy WASPAS for weight estimation and ranking of claims from customers owing to drinking water utility in a firm. Simi and Lazarevi (2021) extended WASPAS to picture fuzzy set for evaluating last mile transport mode within Belgrade. Balezentis et al. (2021) assessed the involvement of stakeholders in sustainable energy development by proposing an integrated BWM-WASPAS method with fuzzy data.

From the review provided above on different extensions of WASPAS, the novelties of this study are as follows:

- WASPAS is a simple and elegant approach for ranking alternatives that helps researchers and practitioners to flexibly use the formulation for different decision problems.
- The classical WASPAS formulation is extended by the diverse frameworks, which considers the linear combination of weighted sum and weight product values of alternatives.
- Fuzzy extension of classical WASPAS is popular in the literature and can be used for solving decision problems.
- Nature of criteria is not considered in these extensions, which can be intuitively seen as a potential information during decision-making process.
- Cumulative ranking is commonly provided by these WASPAS extensions, but personalized ranking based on individual’s data is not well explored.

These insights motivated authors to put forward an improved WASPAS method, which has the following novelty aspect that intuitively adds rationale to the decision-making process.

- Simplicity, elegance, and ease of understanding of the classical WASPAS inspired authors to adopt the method in the present study. Unlike outranking and compromise ranking approaches, the WASPAS has a straightforward formulation and reduces the computational overhead in a feasible manner.
- The proposed ranking method considers nature of criteria during ranking, which was lacking in the other extensions of WASPAS that focused on extending the classical version of the method to different fuzzy variants. As discussed earlier, the nature of criteria intuitively adds rationale to the decision process.
- Also, the proposed ranking gave personalized ordering of alternatives based on the individual’s data along with the cumulative ordering of alternatives, which is obtained by aggregating the rank values of alternatives obtained based on the individual’s data.

Altogether, it can be observed that the improved WASPAS not only takes advantage of the formulation elegance of the classical WASPAS, but also provides an intuitive rationale in the decision process by taking into consideration the nature of criteria and formulating a personalized ranking algorithm that can provide ranking based on the individual’s viewpoint and cumulative context as well.

This section provides a novel extension to the WASPAS approach under a fuzzy environment. From the review conducted above, certain inferences can be made, such as *(i)* WASPAS is a popular method for ranking, *(ii)* nature of criteria is not taken into consideration, and *(iii)* personalized ranking with fuzzy data based on individual expert preference is an open area for exploration.

Driven by these inferences, an improved WASPAS procedure is put forward, which attempts to address these issues. A stepwise procedure is provided below:

*Step 1:* Form $t$ matrices of $r \times q$ order by considering rating from experts of $r$ alternatives over $q$ criteria. Preferences were linguistically obtained from each expert.

*Step 2:* Fuzzy numbers associated with the terms are used for calculating the parameters of improved WASPAS. Apply Eq. (5) to transform data into a homogeneous form by considering the nature of criteria for rational decision-making.

5 $$ tx_{{ij}}^{l}  = \left\{ {\begin{array}{*{20}c}    {1 - \mu _{{ij}}^{l} forj \in cost}  \\    {\mu _{{ij}}^{l} forj \in benefit}  \\   \end{array} ,} \right.  $$

where $tx_{ij}$ is the transformed value.

*Step 3:* Calculate the weighted sum and weighted product values for each option based on the data given by each expert by using Eqs. (6)-(7).

6 $$ U_{i}^{l} = \mathop \sum \limits_{j = 1}^{q} \left( {w_{j} .tx_{ij}^{l} } \right) $$

7 $$ D_{i}^{l} = \mathop \prod \limits_{j = 1}^{q} \left( {tx_{ij}^{l} } \right)^{{w_{j} }} $$

where $U_{i}^{l}$ and $D_{i}^{l}$ are the weighted sum and product values calculated for each alternative based on data from each expert.

*Step 4:* Estimate the total rank for each alternative based on individual preferences and calculate the final ordering based on the fusion process. Equations (8)-(9) are applied for obtaining the values.

8 $$ UD_{i}^{l} = \gamma U_{i}^{l} + \left( {1 - \gamma } \right)D_{i}^{l} $$

9 $$ UD_{i}^{net} = \mathop \prod \limits_{l = 1}^{t} \left( {UD_{i}^{l} } \right)^{{\lambda_{l} }}, $$

where $\lambda_{l}$ is the importance value associated with expert $l$.

Finally, it can be noted that Eq. (8) gives rank values of alternatives based on individual expert’s data, while Eq. (9) provides the holistic ordering of alternatives. The proposed ranking procedure has the following merits: *(i)* It considers the nature of criteria during ranking; *(ii)* It considers both the criteria and experts’ importance values; and *(iii)* It also offers the ordering of alternatives from both the individual’s viewpoint and holistic perception. These claims add to the novelty of the procedure.

## Case study

**Fig. 2:**
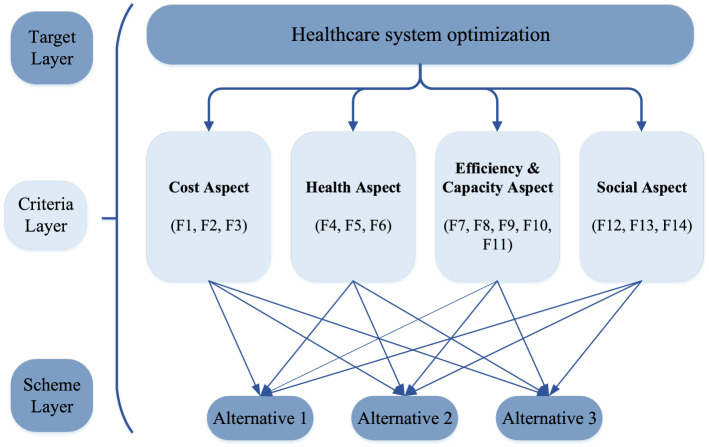
The hierarchical setting of alternatives and criteria for the decision problem

This section presents a real case example of healthcare systems prioritization to manage pandemic situations. Based on the literature reviews, authors identified fourteen potential criteria that could be broadly categorized into four groups, namely cost aspect, health aspect, efficiency & capacity aspect, and social aspect. A detailed explanation of these fourteen criteria is provided in Sect. 3. Four experts form a panel. They rate these criteria for determining the weights (importance) of criteria. Three alternatives are using technological (such as wearable) devices for monitoring and diagnosis of patients to minimize the visit duration, specifying days for handling chronic diseases- (Staff/Patient/Resource Scheduling), and subsidy for the staff for shift days based on the number of incidents. These three alternatives are rated by each of the four experts based on the fourteen criteria. Likert scale rating with nine grades is used for rating whose corresponding fuzzy number obtained from the singleton grading is given in Table 1. As mentioned earlier, fuzzy numbers are a simple and elegant form of preference information that not only supports rational/practical decision-making but also mitigates subjective randomness. Inspired by the simplicity and flexibility of fuzzy values, authors use the same in the current study. A hierarchical view of the problem under consideration is depicted in Fig. 2, which provides readers a clear understanding of the set of criteria within each category and the set of alternatives that the model attempts to prioritize based on the data from experts over these criteria.

**Table 1:** Rating terms with their fuzzy form (Nguyen, 2016)

Linguistic terms	Fuzzy numbers
Extremely low	0.1
Very low	0.2
Moderately low	0.3
Low	0.4
Neutral	0.5
High	0.6
Moderately high	0.7
Very high	0.8
Extremely high	0.9

### Experimental results

The authors prepared the questionnaire and revised the same based on the advice from the experts, who potentially took part in sharing the valuable preferences over criteria and alternatives. The questionnaire was circulated through email to the experts for data collection. For the ease of representation, authors denote $E$ as a set of experts, $R$ as the set of alternatives, and $F$ as the set of factors/criteria. Steps for the selection of a suitable alternative are given below.

*Step 1:* Obtain four preference matrices of order $3 \times 14$ where three alternatives are rated linguistically based on fourteen criteria. Table 1 depicts the Likert scale rating and the corresponding fuzzy number associated with the terms. Nine grading scheme is adopted in the present study. Experts are encouraged to share their views through these grades, which results in decision matrices from experts that are of form $3 \times 14$.

It may be noted that values in Table 1 are considered as preference values when alternatives are rated based on criteria and importance values when experts rate criteria.

Tables 2 presents the linguistic rating information from each expert that attempts to rate each alternative over a set of criteria. Terms specified in Table 1 are used for rating and rational selection of the alternatives, the fuzzy number associated with the term is used. We can observe that each expert provides her/his rating on the alternatives based on the criteria. These linguistic terms are transformed to their respective fuzzy value during the decision process.

**Table 2:** Rating from experts – alternative to criteria in the linguistic form

Experts	Alt	Criteria													
		F_1_	F_2_	F_3_	F_4_	F_5_	F_6_	F_7_	F_8_	F_9_	F_10_	F_11_	F_12_	F_13_	F_14_
Expert 1	R_1_	VL	ML	VL	EL	VH	EH	VH	EH	VH	EH	VH	H	MH	EH
	R_2_	H	MH	ML	L	ML	L	H	MH	L	VL	MH	VH	H	MH
	R_3_	L	EL	VH	H	H	VH	MH	VH	N	N	H	H	N	VH
Expert 2	R_1_	EL	L	EL	VL	MH	VH	EH	MH	EH	VH	EH	MH	H	VH
	R_2_	N	VH	N	H	ML	ML	MH	N	N	ML	H	EH	L	MH
	R_3_	ML	VL	MH	MH	N	MH	VH	H	H	H	N	N	N	MH
Expert 3	R_1_	EL	H	EL	EH	L	EH	L	MH	H	EH	VL	L	H	H
	R_2_	L	N	N	EH	EL	H	MH	H	MH	N	EH	H	EH	EH
	R_3_	N	EL	ML	H	L	MH	H	N	H	N	L	N	VH	VH
Expert 4	R_1_	ML	ML	ML	VL	N	H	H	H	H	EH	ML	VL	N	N
	R_2_	VH	H	L	L	EL	N	EH	EH	EH	H	VH	N	MH	VH
	R_3_	L	EL	ML	EL	ML	L	N	MH	VH	N	H	ML	H	H

*Step 2:* Obtain opinions from experts on each criterion that leads to a matrix of $4 \times 14$ that are also linguistically provided. Based on Table 1, the fuzzy values to the terms are associated.

**Fig. 3:**
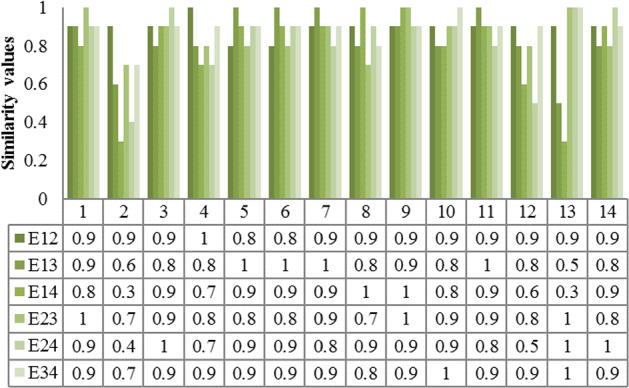
A similarity measure for each pair of expert preferences (X axis – labels 1 to 14 denote 14 criteria considered in the study)

Table 3 presents the importance values provided by each expert on each criterion. This is used by the procedure proposed in Sect. 4.1 for estimating the weights of criteria/factors. Figure 3 shows the relationship between the preferences provided by each expert for the 14 criteria. Since there are four experts, six ordered pairs are possible, which are depicted as E12 to E34 in Fig. 3. For an instance, E12 denotes the preference relationship between experts $E_{1}$ and $E_{2}$ $\left( {E_{1} ,E_{2} } \right)$. Similarly, other combinations include preference relationship between $\left( {E_{1} ,E_{3} } \right)$, $\left( {E_{1} ,E_{4} } \right)$, $\left( {E_{2} ,E_{3} } \right)$, $\left( {E_{2} ,E_{4} } \right)$, and $\left( {E_{3} ,E_{4} } \right)$. These are depicted as legends in Fig. 3. In the formulation, we had surveyed all pairs, and similarity of unity means that two experts provided the same preference value for a particular criterion. To further clarify, let us consider the (1,1) position, which yields a value of 0.9 indicating that the preference relationship of $E_{1}$ and $E_{2}$ with respect to factor $F_{1}$ is 0.9. Other values are interpreted in the same fashion. Intuitively, we could infer that if all preferences for a criterion are the same, then the mean of the measure will be equal to unity. The distance measure will be zero leading to a similarity value of one for all ordered pairs. Equation (2) calculates the distance between opinions by using the Manhattan distance measure. From Eq. (3) we can observe that the consistency value is given as 0.9818, 0.9000, 0.9818, 0.9640, 0.9785, 0.9785, 0.9818, 0.9714, 0.9882, 0.9785, 0.9818, 0.9474, 0.956, and 0.9785, respectively. These values are calculated based on the similarity values from Fig. 3. By applying Eq. (4), the consistency vectors are normalized and the weight of each criterion is calculated as 0.072, 0.066, 0.072, 0.071, 0.0721, 0.0721, 0.0723, 0.0716, 0.0728, 0.0721, 0.0724, 0.0698, 0.0704, and 0.0721, respectively.

**Table 3:** Importance values from experts on each criterion

Experts	Criteria													
	F_1_	F_2_	F_3_	F_4_	F_5_	F_6_	F_7_	F_8_	F_9_	F_10_	F_11_	F_12_	F_13_	F_14_
E_1_	EH	VL	VH	EH	L	H	MH	MH	VH	EH	H	VH	EH	N
E_2_	VH	ML	MH	EH	H	L	H	VH	MH	VH	MH	EH	VH	L
E_3_	VH	H	H	MH	L	H	MH	N	MH	H	H	N	L	ML
E_4_	H	EH	MH	H	N	N	VH	MH	VH	H	N	L	VL	L

*Step 3:* Determine parameters of improved WASPAS that aid in the ordering of alternatives. Table 4 provides the values for weighted sum/product and total ranks.

**Table 4:** Parameters of improved WASPAS based on indivial experts' data

Experts	Alternatives	Experts	Alternatives	$U_{i}^{l}$	$D_{i}^{l}$	$UD_{i}^{l}$	Experts	Alternatives	$U_{i}^{l}$
Expert 1	R_1_	0.765	0.727	0.746	Expert 3	R_1_	0.731	0.66	0.696
	R_2_	0.551	0.518	0.534		R_2_	0.548	0.483	0.513
	R_3_	0.591	0.557	0.573		R_3_	0.634	0.618	0.626
Expert 2	R_1_	0.773	0.747	0.76	Expert 4	R_1_	0.765	0.755	0.76
	R_2_	0.523	0.486	0.504		R_2_	0.557	0.513	0.535
	R_3_	0.606	0.577	0.591		R_3_	0.684	0.671	0.678

Table 4 presents the weight sum/product and net rank values of alternatives based on preferences from an individual expert. These values are calculated by using Eqs. (6)–(8). From the $UD_{i}^{l}$ value, the ordering is determined as $R_{1} \succ R_{3} \succ R_{2}$ for all four experts. Based on these rank vectors and experts’ reliability values as 0.3, 0.2, 0.2, and 0.3, respectively, the aggregated rank value is calculated as 0.7425, 0.5241, and 0.6176, respectively for $R_{1} ,R_{2} ,R_{3}$ with ordering as $R_{1} \succ R_{3} \succ R_{2}$.

## Sensitivity analysis

**Fig. 4:**
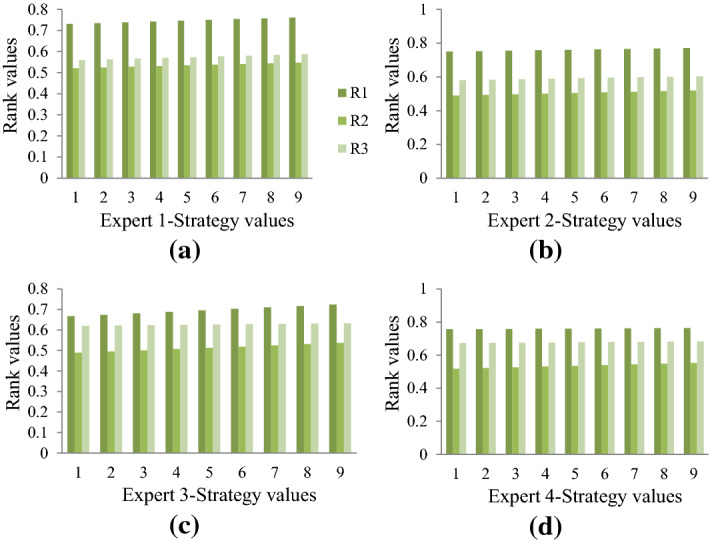
Sensitivity analysis of strategy values with biased weight model and individual expert’s data.

**Fig. 5:**
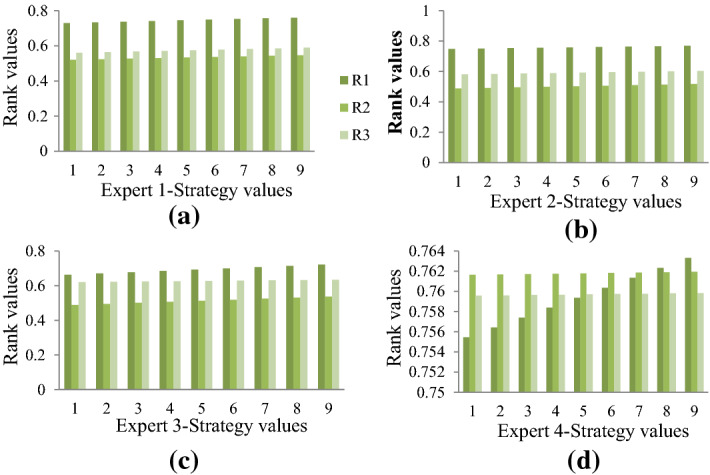
Sensitivity analysis of strategy values with unbiased weight model and individual expert’s data

The proposed framework is validated by altering weight values of criteria to understand the effect of change of weight values on the ordering of alternatives. In this section, a comprehensive inter/intra sensitivity analysis is performed that varies weights of criteria and the strategy values in a stepwise manner. Figures 4 and 5 show the inter/intra sensitivity for biased and unbiased weights of criteria. Biased weights are obtained by applying the procedure put forward in Sect. 4.1 and unbiased weights assign equal weights for all criteria, and individual expert’s data.

The two figures depict the inter/intra sensitivity analysis that alters weights of criteria and strategy values. Figure 4 (a) to (d) depict the rank values for varying strategy values with biased criteria weights obtained by applying the procedure in Sect. 4.1. Similarly, Fig. 5 (a) to (d) depicts rank values when strategy values are altered with unbiased weights. The four figures in Figs. 4 and 5 present the rank values of alternatives based on the data from each expert. It can be inferred that the proposed work produces ordering that is intact in both inter/intra sensitivity analysis. Figure 4d and Fig. 5d produce different ordering, which can be intuitively understood owing to the change of weight values of criteria. From the proposed work, the ordering of alternatives based on different expert data is the same, which shows the data consistency and model robustness.

The holistic rank values calculated for each alternative by using Eq. (9) are given by 0.742, 0.524, and 0.618, and the ordering based on the proposed model is $R_{1} \succ R_{3} \succ R_{2}$. Rank values of alternatives when unbiased weights are used is given by 0.741, 0.582, 0.640 with ordering as $R_{1} \succ R_{3} \succ R_{2}$. This infers that the model is highly consistent and robust even after adequate changes are made to the weights of the criteria. Furthermore, it can be inferred that the broadness of rank values produced by the proposed work (biased rank model) is significantly higher than the unbiased rank model, which gives evidence for the urge to have rational approaches for calculating weights of criteria (Kao, 2010). The broader rank values allow experts to efficiently plan their backup for critical situations.

Further, the proposed model is compared with Agarwal, et al. (2020) to realize the strength of the framework. Table 5 presents the rank values of alternatives obtained based on each expert’s data. From the values, the ordering obtained differs from that of the proposed model and the broadness of values is also less. This provides evidence that the proposed improved WASPAS produces a rational ordering of alternatives by not only considering perception, but also the nature of criteria.

**Table 5:** WAPAS approach parameters with fuzzy data (Agarwal et al., 2020)

Alternatives	Experts				
	E_1_	E_2_	E_3_	E_4_	E_5_
R_1_	0.585	0.558	0.48	0.438	0.511
R_2_	0.502	0.526	0.598	0.596	0.552
R_3_	0.571	0.541	0.501	0.414	0.5

## Comparative analysis

This section brings out the efficacy of the proposed model by comparing the framework with existing fuzzy models for decision-making. Apart from the close counterpart (Agarwal, et al. (2020)’s model), other models, namely Alkan and Albayrak (2020)’s model, and Dhiman and Deb (2020)’s model, are taken for comparison. It must be noted that these models actively adopt a fuzzy environment for preference information. Bot theoretical and numerical comparison is performed to determine the efficacy of the proposed work. In the theoretical context, the efficacy of the proposed work is given in Table 6 based on the feature/characteristic comparison.

**Table 6:** Characteristics summarization of different fuzzy models

Factors	Proposed	Agarwal, et al. (2020)	Alkan and Albayrak (2020)	Dhiman and Deb (2020)
Dataset	Fuzzy numbers			
Nature of criteria	Yes—considered	No—not considered	Yes—considered	Yes—considered
Inter/Intra sensitivity	Performed	Not performed	Not performed	Not performed
Importance of experts	Considered during weight calculation and ranking	Not considered during weight calculation and ranking Not considered during weight calculation and ranking	Not considered during weight calculation and ranking	Not considered during weight calculation and ranking
Personalized ranking	Obtained	Not possible	Not possible	Not possible

Certain novelties that can be inferred are:

- Adhering to the claim of Kao (2010), weights are not only calculated methodically, but also the reliability measure associated with an expert is being considered in the formulation. Cronbach’s measure presented in the present work calculates weights methodically, by taking into consideration the consistency of experts in preference sharing through a comprehensive similarity calculation. Intuitively, the criterion that yields high consistency from the experts’ perception gets high weights, signifying its high importance in the decision process.
- Further, the WASPAS method is extended to consider the nature of criteria during ranking by adopting a transformation procedure that homogenizes the preference information for rational decision-making.
- Also, the sense of personalized ranking is formulated in the present study, by considering data from an individual for determining the ordering of alternatives, which are finally fused for obtaining the holistic ordering of alternatives.
- Further, the proposed work is inferred to be robust even after weights of criteria and strategy values are altered through sensitivity analysis.
- Finally, it is observed that the model is consistent with the extant decision models based on the Spearman correlation.

**Fig. 6:**
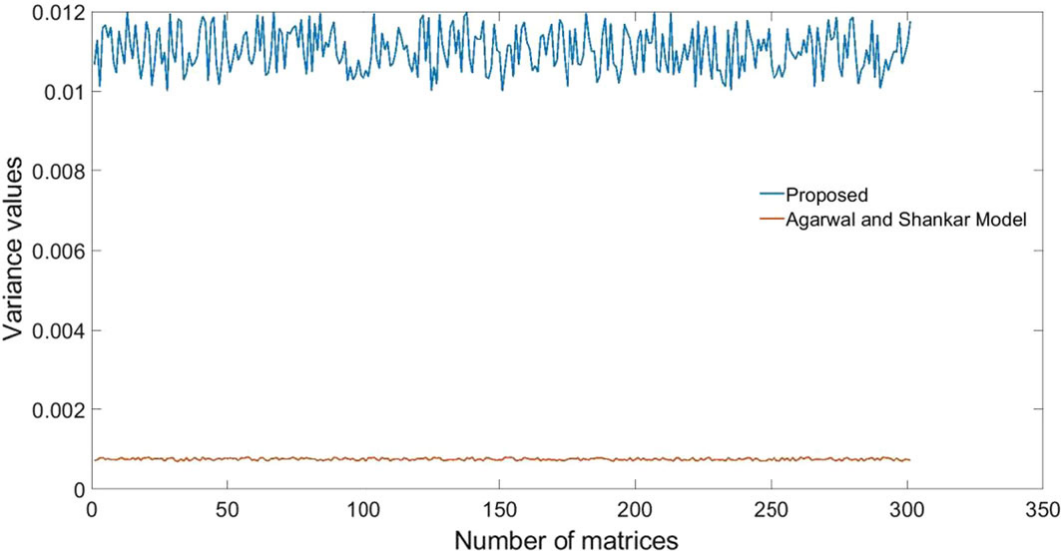
Discriminative/broadness test for the proposed and existing model with fuzzy data.

Besides the theoretical merits of the proposed work, the numerical merits are also investigated. For this we take consistency and broadness or ranks as two measures in this study. For determining the consistency of the proposed work, the ranking is obtained as: $R_{2} \succ R_{1} \succ R_{3}$ for Agarwal, et al. (2020)’s model and Dhiman and Deb (2020)’s model and $R_{1} \succ R_{3} \succ R_{2}$ for Alkan and Albayrak (2020)’s model. Spearman correlation is applied to these orders. The correlation measure indicates that the proposed model is consistent with existing models with Spearman coefficient values as 1.0, 0.50, 0.50, and 1.0, respectively. Further, the efficacy in terms of broadness and discrimination power of the framework is validated by conducting a simulation experiment with 300 matrices of 3 by 14 order. Rank values of alternatives in each matrix are determined by using the proposed model and model Agarwal & Shankar (2020)’s model and the variance is determined for each rank vector. Figure 6 shows the broadness measure of models and results infer that the proposed model outperforms the existing model in terms of the discrimination power, allowing experts to effectively plan backups during crucial situations. From the figure it can be seen that there is approximately (on an average) ten times better variability/discrimination of alternaitves from the proposed work in comparison to the counterpart.

In a nutshell, it is observed that the proposed work adds value both from the theoretical and numerical perspectives. Uncertainty is handled by mitigating human intervention in the decision process, which might cause inaccuracies and subjectivity. A sense of personalization is also provided by the proposed work by considering the preferences of each expert individually. Furthermore, in the numerical context, authors infer that the model proposed is robust to both the alteration of weights of criteria as well as strategy values of experts. Also, the proposed work is consistent with the extant models in terms of ordering of alternatives. The proposed work also produces broad and sensible rank values compared to the counterpart that helps in effective backup management.

## Result and discussion

The three alternatives are prioritized based on the responses. According to the findings, the subsidy for the staff alternative is regarded as the least advantageous. The following advantageous one is the specifying days for patients with chronic diseases. Finally, among the other options, using technological devices is the best.

Among the alternatives, specifying dates and providing an online system is the least effective way. Diseases such as hypertension, chronic ischemic heart disease, and nonspecific lung disease are known as chronic diseases. Also, an increasing number of elderly people suffer from over one (Schellevis et al., 1993). According to the 2011 World Health Organization Global Status Report, chronic diseases account for 63% of deaths (Terzic & Waldman, 2011). Chronic diseases are dangerous in everyday life, but unfortunately, it is much more dangerous during pandemics. So policy-makers should pay regarding the patients with chronic disease.

Subsidy options are seen to be the second most advantageous alternatives to prevent collapses during the pandemic. The financial results of pandemics affect everyone else, including medical staff. On those days, it is critical to creating a financially safe environment for those who were affected the most. Giving financial support during the pandemic can help increase motivation. If the doctors were given assistant doctors to their side for help, this can help them for busy days. Divide the number of patients between professionals rather than compel them to one.

At the point of decision making, decision-makers should try to integrate technological developments into healthcare systems to reduce negative aspects of pandemics.

## Managerial policy implication

Infectious diseases which are pandemics have shaped human civilization in almost every aspect. Their effects sometimes lasted for centuries and wiped out populations. A recent outbreak was COVID-19, which affects our daily lives. Governments attempted to get over the situation with some solutions as lockdowns. Using optimization tools is one of the most powerful solutions to prevent collapse and the number of infected patients. Using technological devices is the most advantageous alternative in avoiding disorder and spreading the rate of pandemics. Governments should evaluate the alternatives to enhance the usage of optimization tools related to healthcare. Telehealth interviews are accessible and a barrier that limits the spread of pandemics. Pandemics are spreading from person to person. So, people be careful about providing social distancing. Enhancing and promoting online healthcare systems can create a healthy and socially distanced environment.

**Fig. 7:**
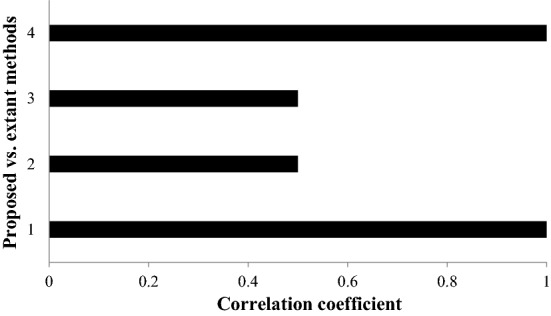
Spearman correlation for consistency analysis—proposed vs. extant models

Some advantages of the proposed integrated approach in terms of theoretical and methodical perspectives are listed as follows: (i) Weights are calculated methodically be properly understanding the interrelationship among the preference vectors by experts, which effectively reduces biases and inaccuracies. Further, the imrprovement in the WASPAS approach for ranking alternatives rationally is another advantage from the theoretical perspective, which allows practitioners to take advantage of the simplicity of WASPAS and incorporate formulation to understand nature of criteria and perform personalized ranking of alternatives along with cumulative ranking. (ii) The proposed integrated approach also us unaffected by alteration of weights of criteria and strategy values of experts inferring the robustness of the model. Also, in the methodical perception, the proposed approach has higher discrimination ability compared to the close counterpart and method is consistent with the state-of-the-art decision approaches. These can be inferred from Figs. 5, 6, and 7.

## Conclusion

It is seen that using technological devices is the most advantageous alternative to prevent collapses during pandemics. With an enhanced online health system and wearable products, patients do not have to go to the hospital during pandemics. Through video discussions with health specialists, telemedicine can help diagnose (Smith et al., 2020). Similarly, during an infectious disease epidemic, it can help patients access the health care system. Beyond the emergency, telemedicine adoption has been weak and scattered. Many attempts have been made to increase the regular use of telemedicine, with fair results (Peddle, 2007; Smith & Gray, 2009). The low adoption of the services is primarily due to reluctance to use telemedicine. Constant telemedicine use contributes to more sustainable treatment modalities (Cottrell et al., 2018). It must be integrated into training and skills to ensure that the health staff is telemedicine services. It is critical to incorporate video conferencing into the curriculum and to need accreditation (Edirippulige & Armfield, 2017).

Wearables are in tremendous growth, especially in the healthcare industry (Islam et al., 2015). Wearable sensor technology provides a chance to shift predicting from a onetime figure demonstrates an objective method for prodromal-stage diagnosis of infectious disease's progression. With wearable devices, individuals' health conditions can be observed continuously. Wearables are simple and capable of continuous tracking with minimum user involvement. Additionally, they are effective in inpatient or distant situations, offering a comfortable examination of patients (Channa et al., 2021).

The proposed model adds value to the healthcare domain by rationally prioritizing alternative healthcare systems in concern to the pandemic outbreak. The elegance and flexibility of fuzzy data, along with its ability to mitigate subjective randomness motivated authors to put forward a novel model that integrates methods for criteria weight calculation and prioritization of alternatives. Attitudinal Cronbach’s measure is proposed for weight estimation that not only considers data consistency, but also the attitudinal characteristics of experts. The proposed weight calculation method could effectively capture preference relationships from experts’ viewpoints and model weights based on a methodical scheme with appropriate capturing of hesitation in preferences/opinions. As discussed earlier, the higher similarity among preference vector from experts indicates that the views are similar with less variability in views. Furthermore, the WASPAS method is extended to consider nature of criteria and also provide personalized ranking of alternatives based on individual expert’s data along with holistic ordering of alternatives. This improvement to the classical WASPAS gives rationale in two-folds: (i) the ranking algorithm can now take nature of criteria into consideration, which is essential aspect in systematic ranking of alternatives; and (ii) individual ranking along with holistic ranking not only gives final ordering of alternatives in a cumulative sense, but also gives ordering from each expert’s viewpoint that offers a personalized decision in the process. Intuitively, the data integrity and intent is retained during individualistic opinion based ranking, unlike the models that adopt fused opinions where there is certain loss of information. Rationally, authors attempt to mitigate that loss in the present study.

Sensitivity analysis of weights and strategy values from experts reveal that the proposed model is highly robust even after altering these values. These are seen as inter/intra sensitivity analysis from Fig. 4 and Fig. 5. Besides, the discrimination power of the proposed model is significantly high compared to its close counterpart approach, indicating that the proposed model can effectively allow experts to plan backups during crucial situations (see Fig. 6). Also, the consistency of the model is evident from the correlation coefficients obtained for proposed versus other existing models (see Fig. 7). From the statistical perspective, these advantages are inferred for the proposed work and from the theoretical perspective, the proposed model (i) mitigates human intervention by calculating parameter values rather than direct assignment; (ii) captures preference relationship from experts during criteria weight determination; and (iii) ranks alternatives based on the personalized views from each expert by considering nature of criteria along with the holistic ranking for rational decision-making.

Certain shortcomings of the proposed model are: *(i)* reliability values of experts are directly acquired rather than methodical estimation in this study that might cause subjectivity and biases in the decision process; *(ii)* though personalized ranking is obtained, query based ranking is not formulated that would result in customized ordering of alternatives based on the demand vector; and *(iii)* data is assumed to be complete, which is not practical in many real-time situations that causes non availability of values resulting in sparse data space.

As part of the future direction towards research, the shortcomings mentioned above could be addressed. Besides, the developed model can be used for barrier assessment in technology adoption, sustainability adoption, and lean/agile paradigm adoption. It is also well known that the preference elicitation in the decision process is a complex and dynamic event that must be well modeled by using higher dimension data spaces that aids in better modeling of uncertainty. The proposed model could also be extended to other medical areas such as location identification, selection of medical waste treatment options, and staff selection for medical centers. In addition, newer orthopair based frameworks with game theory models can be used for the healthcare system ranking.

With technological improvements, it is possible to have a sustainable healthcare system in each condition. They can help diagnose earlier. Those improvements are essential for the diagnosis of chronic diseases. Healthcare systems integrated technology is a vital step for medical advancement.

## Appendix

Table 7 is presented below that describes the symbols used in the equations in the present paper.

**Table 7:** Symbols and its meaning

Symbols	Meaning
$t$	Number of experts
$q$	Number of criteria
$j$	Index for criteria
$d_{j} \left( {a,b} \right)$	Distance between any two experts $a$ and $b$ with respect to criterion $j$
$\mu_{a} ,\mu_{b}$	Membership grades from any two experts $a$ and $b$
$\rho_{j} \left( {a,b} \right)$	Similarity between the values from two experts $a$ and $b$ with respect to criterion $j$
$\beta_{j}$	Cronbach coefficient of criterion $j$
$w_{j}$	Weight of criterion $j$
$r$	Number of alternatives
$tx_{ij}^{l}$	Transformed membership grade from expert $l$ to rate alternative $i$ based on criterion $j$
$\mu_{ij}^{l}$	Membership grade from expert $l$ to rate alternative $i$ based on criterion $j$
$l$	Index for expert
$i$	Index for alternative
$U_{i}^{l}$	Weighted sum value from expert $l$ for alternative $i$
$D_{i}^{l}$	Weighted product value from expert $l$ for alternative $j$
$UD_{i}^{l}$	Net rank value for the data from expert $l$ associated with alternative $i$
$UD_{i}^{net}$	Aggregated rank value of an alternative $i$
$\gamma$	Strategy value
$\lambda_{l}$	Weight value of expert $l$
